# Risk Management Through an “Activity Contradictions” Lens: Exposure to Low Doses of Radiation in Nuclear Medicine

**DOI:** 10.3389/fmed.2019.00228

**Published:** 2019-10-24

**Authors:** Romain Lonceint, Françoise Bodéré, Bénédicte Geffroy

**Affiliations:** ^1^IMT Atlantique, Laboratoire d'Économie et de Management Nantes-Atlantique, Nantes, France; ^2^CHU Nantes, Centre de Recherche en Cancérologie et Immunologie Nantes-Angers, Nantes, France; ^3^Nuclear Medicine, University Hospital, Nantes, France; ^4^CRCINA, INSERM, CNRS, Université d'Angers, Université de Nantes, Nantes, France

**Keywords:** risk management, activity contradictions, nuclear medicine, care, radiation protection

## Abstract

Risk management is a major concern for health organizations. In hospitals, it concerns both medical and occupational risks, particularly those related to exposure to ionizing radiation. Medical personnel represent 70% of workers exposed to ionizing radiation. The highest doses in the order of a few mSv are recorded in nuclear medicine departments. Nuclear medicine health professionals are thus exposed, in the context of their work activity to daily low doses—though their effects remain uncertain. In the face of this uncertainty, the precautionary approach prevails in the field of radiation protection. Thus, health professionals are called upon to treat the patient while protecting themselves from exposure to low doses of radioactivity. This research aims to understand the relationship of health professionals to the risks of exposure to low doses and how they combine the logic of patient care and cure with that of self-protection. It is based on a qualitative study of two embedded cases carried out in two units of a nuclear medicine department at a university hospital, combining two data collection methods: 23 interviews with various health professionals in the department and 10 weeks of observations of the work activity of these same professionals. The analysis of the data shows the coexistence of care/cure and radiation protection logics to be a source of contradictions for nuclear medicine professionals. Analysis of the results focuses on the identification and characterization of the different forms of contradictions inherent in working in the nuclear medicine department. The results show that the intensity of these contradictions varies in line with four factors: phases (preparation, administration, patient installation, and examination); type of medical act; patient behavior and characteristics, and type of professionals. Finally, the results set out the different types of responses provided by health professionals in order to regulate these contradictions. These risk regulation strategies differ according to occupational groups and their relationship to risk.

## Introduction

Risk management is a major concern for health organizations. In hospitals, it concerns both medical and occupational risks, in particular those related to exposure to radioactivity. While radiotherapy accidents such as the one that occurred at Épinal Hospital (where several patients were overexposed between 2001 and 2006) have contributed to higher-profile media visibility for this type of risk to patients, the risk associated with exposure to radioactivity also concerns hospital staff. Indeed, health professionals represent 70% of workers exposed to radioactivity, making this occupational risk a subject of interest for health organizations. Occupational exposure to radioactivity in the medical sector generally falls within the domain of low doses, for which the risks are not known (1). This is therefore a situation of uncertainty in the sense that no “causal explanation system” (2) has been established between exposure to radioactivity for doses below 100 mSv and the appearance of pathologies. However, this lack of a causal link between exposure to low doses and the appearance of pathologies does not mean that the risk does not exist. Thus, applied to the field of low doses of radioactivity, radiation protection is based on a logic of prudence and precaution since it applies to hypothetical risks (3).

In addition to these uncertainties relating to radioactive risks, nuclear medicine professionals also find themselves confronted with medical uncertainties. A number of studies in the field of medical sociology (4, 5) have highlighted the finding that uncertainty is inherent in any medical practice. This research has shown that the knowledge and techniques of medicine and doctors are only ever less than perfect, and that the result of a treatment or examination can never be known *a priori*. The existence of these medical uncertainties means that, in the benefit/risk ratio in medicine, the result is always an expected benefit. Nuclear medicine does not escape the problem of medical uncertainties that directly affect the healthcare (and thus, the medical benefits) of imaging examinations and treatments.

In addition, the implementation of radiation protection measures may lead health organizations to believe that the issue of risks associated with radioactivity is controlled. The absence of personal protective equipment (or circumvention of radiation protection rules) is then interpreted as a failure to comply with recommendations. However, this ignores the fact that social science research has long shown, first, that there is a link between risk representations and professional practices (6) and second, that these practices (perceived as deviations from the rules) reveal that in the background lies a lack of consideration of the activity as it takes place, including all of its contradictions and requirements (7). Occupational risk management based on the application of radiation protection measures thus goes beyond the sole question of technical and scientific understanding of the risk, in that it is also marked by different professional logics and the situated nature of work practices. This is why we consider occupational risk in the context of the work activity, that is, the practical accomplishment of the activity (8).

The research focuses on occupational exposure to radioactivity in the nuclear medicine sector. This is a medical specialty that includes all applications of radiopharmaceuticals for diagnostic and therapeutic purposes. However, the use of radiopharmaceuticals in imaging examinations or therapeutic procedures is a source of daily occupational exposure to radioactivity. The challenges of exposure to low doses call for an interest in the management of a hypothetical occupational risk in nuclear medicine, as distinct from the nosocomial infections that are a proven risk faced by health professionals (9). The analysis of work activity in nuclear medicine reveals the coexistence of two potentially contradictory logics of action, namely the logic of patient care in the context of diagnostic or therapeutic medical procedures and the logic of self-protection against the possible risks associated with exposure to low doses of radioactivity. This leads us to analyze occupational risk management in nuclear medicine from the point of view of work contradictions. Thus, this research aims to answer a 2-fold question:

1. What are the forms of contradictions between patient care and radiation protection in nuclear medicine?
2. What methods of regulating these contradictions are implemented by health professionals?

In order to answer this question, the research is based on a qualitative survey conducted in two nuclear medicine units and combining semi-directive interviews with *in situ* observations (Box 1).

Box 1 **Methodological framework and survey field.**

Since the knowledge project focuses on perception of the risks of exposure to low doses and its management methods, we have opted for a qualitative research methodology that is characterized by a comprehensive approach. Indeed, as highlighted by Mays and Pope, “The goal of qualitative research is the development of concepts which help us to understand social phenomena in natural (rather than experimental) settings, giving due emphasis to the meanings, experiences, and views of all the participants” [(10), p. 43]. In opting for such a methodology, we seek to understand how actors think, speak and act in relation to a particular context (11).

We therefore opted for a case study to gain an in-depth understanding of this field of investigation (12). Since our objective was to understand the risk relationship of professionals to low-dose exposure through their radiation protection practices (13), we sought to collect data according to a number of aspects such as: locations, persons (actors) and activities (14).

The survey was conducted in two units of a nuclear medicine department of a university hospital. These two services, under the responsibility of a head of department, are located on two separate sites at the university hospital. One of the services (MN1) is specialized in nuclear medicine for therapeutic and diagnostic purposes in the field of rheumatology, endocrinology, pulmonology, and urology, and the other service (NM2) mainly specializes in diagnostic examinations in cardiology.

According to Yin (11), the objectivity of the case study is based on “multiple sources of evidence” (p. 10). There are traditionally six such sources: direct observations, interviews, archival records, documents, participant observation, and physical artifacts (e.g., computer downloads of employees' work). It is this heterogeneity of empirical sources in qualitative research that guarantees its objectivity, because it allows data to be triangulated. Also, we used a double data collection system, combining semi-directive interviews with observation. This system is also well-suited for analyzing the meaning that actors give to their practices and the events with which they are confronted, in particular their social representations, value systems and interpretations of conflict situations, as well as the reconstruction of action processes.

Similarly, unlike a quantitative approach that aims for representativeness and thus allows statistical inference, representativeness in qualitative research is based on the criteria for selecting individuals (which is the maximum diversity of profiles with regard to the problem studied) and on the principle of saturation (which refers to the fact that, as the interviews succeed one another and reveal their lessons, the contribution made by each additional interview will be minimal). Twenty three interviews were conducted with the various categories of professionals in the nuclear medicine department (see Appendix 1).

The content of the interview covered: the person's background; their role and place within the department's activity; the characteristics of their working environment; the perception of exposure to radioactivity in the context of nuclear medicine activity; consideration of radiation protection in working practices; any difficulties encountered in implementing the various radiation protection measures and the sources of risk (socio-organizational factors impacting exposure to ionizing radiation). Other interviews with actors outside the department were also conducted with: an INSERM radiobiologist; a nuclear doctor (member of the National Academy of Medicine); a nuclear doctor and epidemiologist at IRSN; an occupational doctor, and an ASN inspector. All interviews were recorded and transcribed. To study working practices, this data collection system was supplemented by field observation (15) in the context of exposure to low doses. Indeed, the relationship to an uncertain risk and its management methods could not all be identified solely in the light of the actors' discourse, because there may be a gap between what the actors say about compliance with radiation protection measures and what they actually do in terms of risk regulation. This may be explained by the fact that actors may be blind to their own practices (because these are totally internalized) or that some practices developed consciously by actors are difficult to verbalize. Observation allows the researcher to access representations of actors constructed from their own perceptions (16) and update the resources mobilized by actors in their practices. Thus, a total of 10 weeks of observation were carried out in the two nuclear medicine departments. The objective was to deepen understanding of work practices, particularly in the field of radiation protection, as actual work always exceeds the prescribed work. Observation makes it possible to go beyond normative discourses on the risk of exposure to low doses to grasp the meaning of the gap between doctrine and practice. Observation sequences focused on action in situations, making it possible to understand the situated nature of the practices by considering the multiple variables that constitute health professionals' environment. In practice, it was a matter of direct observation of both actions and activity, as well as the collection of elements of informal discourse gleaned here and there, as the researcher's presence in the field allowed. Collection of this data resulted in notes being taken in a notebook, named a “research journal” by Wacheux (17).

The data collected were then subjected to thematic content analysis (18) using N vivo to identify and analyze professional practices for regulating low-dose exposure risks (The main role of this data processing software is to help manage, format and give meaning to qualitative data).

Thus, we sought to characterize the data by mode of collection (interview or observation) and unit (MN1 or MN2). Each interview was analyzed according to the respondent's occupational group (nuclear doctor, cardiologist, manipulator, nurse, etc.) in order to characterize the different professional logics. Then we developed a number of categories and sub-categories of analysis (19) in order to be able to code the different management situations (20), their dimensions and variability. The coding method used is both bottom-up and top-down in the sense that it is based on a combination of “*a priori* coding” from the literature and “emerging coding” that refers to categories of analysis from the field (21). The data processing made it possible to trace facts back to the general proposals.

First, we show that activity in nuclear medicine gives rise to different contradictions as a result of the coexistence of the logics of patient care and of self-protection. In a second step, we describe the methods implemented by the various professional groups to regulate the contradictions that are inherent to the nuclear medicine activity. These are based on differentiated relationships to the risk associated with exposure to low doses of radioactivity. Occupational risk management is thus understood in the light of the contradictions inherent to working in nuclear medicine.

## Nuclear Medicine Activity at the Root of Contradictions Between patient care and Radiation Protection

### The Coexistence of Patient Care and Radiation Protection Logics

Occupational exposure to low doses of radioactivity in nuclear medicine requires the implementation of protection against the risks of contamination and radiation. The work of nuclear medicine health professionals is to provide care to the patient while protecting themselves from radioactivity. Health professionals are thus called upon to jointly manage both patient care and the application of radiation protection rules. In other words, the work activity in nuclear medicine consists of articulating and combining two heterogeneous logics of action, namely patient care and self-protection. Analysis of the activity reveals the coexistence of these two logics of action: “*You have to work fast as you can, and as best you can. So obviously, you have to keep in mind all the protective screening as well. You have to work with leaded shields and the shielded case - but then it's all about trying to be as efficient and as quick as you can”* (Technologist NM1).

On the one hand, the logic of patient care is characterized by the dual nature of the care activity: the cure activity (the provision of care to someone to cure a disease with the aim of eliminating it, to improve the patient's state of health), and the care activity, which is more oriented toward caring for someone and seeking their well-being (22). Care, then, can be understood as the management of a patient as part of a medical procedure requiring both technical-scientific work (handling machines and administering treatments) and expressive-communicative work (informing and reassuring the patient) (23). In this respect, patient care appears to be relational work, with patients and their relatives as well as between health professionals (24). Moreover, as a medical specialty that has an essentially diagnostic focus, the logic of patient care in nuclear medicine is guided by the search for “*the perfect image*,” that is, the production of a quality image, suitable for interpretation by the practitioner in order to establish a diagnosis.

On the other hand, the logic of self-protection or radiation protection seeks to limit the exposure of nuclear medicine health professionals to radioactivity. The logic of radiation protection is based on a number of rules, such as the use of dosimeters, but also on principles such as the ALARA (As Low As Reasonably Achievable) principle. This principle implies integrating into the work activity the triptych “distance, screen, time”: distance refers to the distance from the radioactive source and the use of remote controls of the processes; screen refers to the use of leaded shields when the activity does not allow movement away from the radioactive source; time refers to the duration of exposure which must be reduced as much as possible. This demands rapid execution of gestures and movements (25). The following quotation reflects the logic of radiation protection in nuclear medicine: “*Let's say that working in nuclear medicine, you really have to be extremely vigilant. Vigilance must be constant, saying to yourself all the time: is being there good or bad? And always keep in mind that as soon as you can get away from the patient, away from the source, you must do so. But then, it happens that a child needs to be held. The distance that is our first protection, is the distance from the source, from the patient being injected. You can't necessarily work from behind leaded screens. So, there are the screens, but it's already the distance and then the time, since we're going to try to inject as quickly as possible and when we need to be with the patient to do something, whatever it may be, we're going to try to be as quick as possible, so as not to stay too long with the patient”* (Technologist NM1).

The coexistence of patient care and radiation protection approaches in nuclear medicine leads to contradictions in the activities of health professionals. These contradictions result from a situation of competition over objectives, in which actors are confronted with multiple and divergent objectives. Indeed, health professionals are required to provide patient care from a diagnostic or therapeutic perspective while continuously applying radiation protection principles and rules so as to limit their exposure to radioactivity, related to both the radiopharmaceutical and the patient. Occupational risk thus arises not only from the handling of radiopharmaceuticals, but also from working in the presence of the patient once the radiopharmaceutical has been administered. The patient is therefore both the object of care and a potential source of risk, as one NM1 technologist points out: “*We're handling radioactivity here, it is both the product and the patients - because the patients are radioactive”*.

Moreover, the contradictions between patient care and radiation protection appear to be inseparable from the work activity in nuclear medicine, insofar as health professionals are confronted with a permanent risk of irradiation and contamination, which requires that radiation protection rules be considered throughout the care activity, as expressed by this radio pharmacist of NM2: “*We are all exposed, if we work with ionizing radiation. So as soon as we enter the nuclear medicine department, we are all exposed, we all have a dosimetry”*. The risk is described, then, as inherent to working in nuclear medicine, according to an NM1 technologist: “*Radioactivity is ambient, it's everywhere. Patients become sources, and that's why you can't stay too close to the patient”*.

### The Forms of Contradiction Between Patient Care and Radiation Protection

In addition, contradictions between patient care and radiation protection take two forms, related to the various aspects of patient care. First, there are contradictions between cure activity and radiation protection, reflecting the existence of contradictions between the administration of patient care (including injection of the radiopharmaceutical or positioning the patient under the gamma camera) and the application of radiation protection rules. The following verbatim account allows us to take stock of this first form of contradiction: “*The lady we saw earlier, the one with no legs, we spent a lot of time with her, beside her. So in this case we are much more exposed. With a patient who is autonomous and everything, who is doing very well, we quickly put the tape around the chest, we put it in place, we move away. Well, on the other hand, the one with no legs, who we need to do everything for, to set everything up, we spend time with her. We're more exposed, that's for sure”* (Nurse NM2).

Second, contradictions are expressed between patient care activity and radiation protection, reflecting the existence of contradictions between patient reassurance and the application of radiation protection rules, as the following verbatim account shows: “*If they are a bit anxious, or a bit claustrophobic, you have to stay close, there are people who panic about the camera, about the examination. Sometimes it's the brain [patients undergoing brain scans], you stay next to them to reassure them, so they can see the examination through. To reassure them, to talk, so they can hear that there is someone right there, because it is not easy to go behind a screen, and they feel as though they are alone. So sometimes they are anxious about that, about being alone”* (Nurse NM2).

The analysis also suggests that these contradictions are divided into three forms, relating to the various aspects of radiation protection. First, spatial contradictions, insofar as care implies being with the patient to provide care and reassurance, whereas radiation protection, on the contrary, requires putting the patient at a distance to protect yourself from it. This is reflected in the following verbatim account: “*A child, even if they are strapped down, you still have to be a little more present. Sometimes we have to hold them, touch their cheeks, so that they stay still, to get a good image. When they are babies or children, we have to stay closer to them. We get more doses.”* (Technologist NM1).

Second, there are physical contradictions, since the use of radiopharmaceuticals does not always allow the complete interposition of leaded screens between health professionals and radioactive products, as shown in the words of a radio pharmacist at NM2: “*In the hot lab, this is where we are likely to get the highest dose, because it's where we prepare the radiopharmaceuticals. It's at the extremities - the fingers – that we're vulnerable, because although the armored enclosure protects us at full body level, at the extremities we still have to put our hands in a lot actually, to make the preparations”*.

Third, there are temporal contradictions, because care requires taking the time to provide care and reassurance to the patient, whereas radiation protection requires working quickly to limit the duration of exposure to radioactivity. The following verbatim account allows us to grasp the issue of temporal contradictions: “*You have to reassure them, but it's the same thing, it's a little odd, because you haven't got three hours to spend reassuring them, because your capsule is there, you know, and so what worried me was that once they had been given the capsule there were some who asked questions at that time, and you at that time have a single desire: to get away from there”* (Nuclear Doctor NM2).

In short, we show that working in nuclear medicine is a source of contradictions that arise in multiple forms, related to the various aspects of patient care and to radiation protection. These contradictions, which appear consubstantial with the work activity of nuclear medicine health professionals, demand implementation of the answers we are now endeavoring to present.

## The Regulation of Contradictions Between Patient Care and Radiation Protection in Nuclear Medicine

### Disregarding Radiation Protection in Favor of Patient Care

Contradictions between the logics of patient care and self-protection can be managed by disregarding radiation protection in favor of care. This response to contradictions can be analyzed from the point of view of the division of labor, insofar as those who disregard radiation protection in favor of patient care are also those who are least exposed in their work activity. Indeed, patient management within nuclear medicine units is based on a division of labor between medical personnel (nuclear doctors and cardiologists) and paramedical personnel (technologists and nurses). Doctors carry out consultations and image analysis in order to establish medical diagnosis; however, they neither handle radiopharmaceuticals nor position patients under gamma cameras. Conversely, paramedical personnel proceed with injection of the radiopharmaceutical, positioning of the patient under the gamma camera and reconstruction of the images, prior to their analysis by doctors. Finally, doctors work mainly on the patient's imaged body, i.e., a representation of the body produced by the imaging technique, while paramedical professions work mainly on the patient's physical body (26). This division between work on the imaged body and work on the patient's physical body, which also refers to the separation between interpretative and productive work (27), has a direct impact on levels of exposure to radioactivity of the various professional groups in nuclear medicine. Thus, by working mainly on the patient's imaged body, exposure to radioactivity of nuclear doctors and cardiologists is relatively low, unlike paramedical personnel who work mainly on the patient's physical body. The distribution of work is therefore not only technical, but also concerns exposure to radioactivity. “*We are not exposed to very high doses in the department, at least not the way it is designed - that is, the technologists ultimately spend much more time with patients than we do. We tend to see them before they've been injected, perhaps again afterwards if they wish, but we are not exposed to very high doses”* (Nuclear Doctor NM1).

The division of labor within nuclear medicine units therefore helps clarify disregard of radiation protection in that it also corresponds to a vertical distribution of risk, in which the most irradiating activities are performed by paramedical personnel. In addition, disregard of radiation protection can also be assessed in terms of the risk profile of these actors. Indeed, both doctors and radio pharmacists are challenging the idea that any exposure to radioactivity is a potential source of risk. The existence of risks associated with exposure to low doses is thus put into perspective, as shown in the following verbatim account: “*Ultimately, we are subjected to the ionizing and deadly radiation of our products[laughs]. Ionizing yes, deadly no. Honestly, I don't have a lot of experience, I'm not going to be able to tell you stories like a seasoned pro, but what we were told at our first lecture, what I heard in the workshops, whether it was true of my leaders or even of the other interns, is that on the whole, low doses are not massively risky.”* (Nuclear Doctor Intern NM2).

Occupational risk is considered, then, to be low or non-existent insofar as it has not been identified. According to these actors, the uncertainty associated with exposure to low doses of radioactivity reflects the existence of a negligible risk—or even the absence of risk. The question of evidence and causal relationship appears at the heart of these two interpretations of uncertainty: “*We do have a fairly scientific culture, yet we're still awaiting papers that demonstrate the risk of low doses – or at least, any excessive risk, high enough to be taken into account - because I am slightly inclined to think that risk is a part of life, but I think we have much less risk of dying as a consequence of low doses than from getting run down on the street, or in a car accident on holiday.”* (Nuclear Doctor NM2).

Disregarding radiation protection thus results in prioritization of patient protection over self-protection. As the following verbatim account shows, these actors are more inclined to implement radiation protection principles to protect patients (such as optimization to limit exposure to radioactivity as much as possible), than to protect themselves from radioactivity in the course of their professional activity. Indeed, patient protection (unlike self-protection) is considered an integral part of patient care. More generally, the disregarding of radiation protection in favor of patient care results in the prioritization of the logic of patient care over the logic of self-protection. Indeed, unlike patient care, radiation protection does not appear to be a structuring dimension of these actors' work activity. The operational rules of radiation protection are thus poorly adhered to in work practices, and this is highlighted by the following verbatim account: “*I have no significant exposure in my opinion, so dosimetry is absolutely not a concern, and neither is radiation protection, as far as I am concerned. For me, this is not a concern at all”* (Nuclear Doctor NM1).

Lastly, disregarding radiation protection in favor of patient care allows these actors to manage the contradictions between the logics of patient care and of radiation protection in that it entails removing one of the two logics of action underlying the contradictions from working practice, as shown in the following verbatim account: “*I have never stopped myself from going to move the patient, or if I see that the technologist is struggling to get them on the table, I will go. At no point will I be reminding myself not to take too long. I'm not looking to spend more or less time in the hot lab or next to the syringe or next to the patient. Later on, when you're interpreting, you're always behind the protective glass, so it doesn't make any difference. But yes, if the patient needs moving, or if I see that the syringe has been placed just behind me, I'm not going to stand aside watching someone else make the effort”* (Nuclear Doctor Intern NM2).

### Adapting Radiation Protection to Patient Care Work

Unlike doctors and radio pharmacists, paramedical actors (technologists, nurses and preparers) have to manage the contradictions between the logics of care and of self-protection, by adapting radiation protection to patient care. This response aims to hold the two logics of action together, rather than disregarding radiation protection in favor of patient care. Indeed, paramedical actors seem to show a differentiated relationship to risk, considering that exposure to low doses is likely to have harmful effects, although these low doses are not necessarily harmful to the working group, as these words of an NM1 technologist reveal: “*I don't often admit it, but I am a little afraid of radiation”*.

The uncertainty associated with exposure to low doses is interpreted as a potential source of occupational risk, rather than as an absence of risk. In other words, these actors establish a possible causal link between their occupational exposure to radioactivity and the incidence of adverse effects. This interpretation of uncertainty is based in part on the experience of the actor and the professional group, as this excerpt from the interview shows: “*In the department, three of us had children and all three of us had major problems. It does make you ask questions, after a while. You tell yourself, unlucky, but there are three of us and we've had a lot of problems with our pregnancies, or a lot of pregnancies that didn't make it to full term. So, then we did ask ourselves: wasn't it because of our environment that we've had problems? So, we realized, even though we don't know for sure, maybe it can be a factor, and we should take care of ourselves. We are in charge of working practices that can affect our health”* (Technologist NM2).

Paramedics also point out that the potential risk is not so much from exposure to low doses of radioactivity as from the repeated nature of this exposure. Thus, according to a nurse from NM2: “*There are risks, because it accumulates over time”*. For health professionals, the risk therefore results more from the accumulation of long-term exposure doses, as evidenced by the following verbatim account: “*I think that by the end of a career, there can be concerns. That's why I think a whole career in nuclear medicine…then it's like smoking and lung cancer, you have some that won't, and some that will”* (Technologist NM2).

The relationship between paramedical actors and risk leads them to take radiation protection into account in their work activity, i.e., to act “as if” the risk associated with exposure to low doses of radioactivity were real, even though they know it is only hypothetical. Thus, as the following verbatim account highlights, radiation protection appears to be a structuring dimension of the activity of these actors, in that it should enable them to protect themselves from possible risks: “*There is no risk as long as these measures are respected, otherwise there may be consequences for our bodies. But if the instructions are followed properly, there is no reason to be afraid to work here”* (Technologist NM1).

Our analysis of the data allows us to underline the fact that these actors manage contradictions by adapting radiation protection to patient care in order to hold the two logics of action together at the very source of the contradictions. Insofar as these actors consider that occupational risk results from the accumulation of radiation exposure doses, the adaptation of radiation protection to patient care results in the development of practices for the division of patient care work that are aimed at collectively distributing radiation exposure doses. Adaptation is thus based on the rotation of workstations concerned with the management of routine procedures. To this end, paramedical staff in nuclear medicine units share responsibility for medical procedures. This distribution is based on the introduction of mechanisms for rotating workstations. Indeed, each work schedule is associated with certain predefined tasks in the various examination and therapy rooms. Rotations take place on a weekly basis, since the technologists and nurses change their working hours (and therefore their shifts) each week. These rotation systems allow a distribution of doses of exposure to radioactivity. According to one NM2 technologist, this represents “*dose rotation”*. Indeed, within each unit of the nuclear medicine department, there is a tacit agreement between paramedical staff to balance their levels of exposure to radioactivity. This work organization makes it possible to distribute exposure to radioactivity among technologists and nurses, insofar as the various workstations are more or less radiant, as shown in this verbatim account: “*We must all rotate our work across different rooms and if we do so, we are less irradiated too, since there are some examinations, there are days when, depending on your working hours, your schedule and your room, you get more or less radiation. So that's another benefit of rotation - you're not always irradiated in the same way”* (Technologist NM2).

In addition, this dose distribution is based on an organization of work established independently by the technologists and nurses of the nuclear medicine units. These actors use what room for maneuver they have to organize their patient care work internally and establish a balance in terms of exposure to radioactivity. Indeed, several technologists and nurses point out that this job rotation system is at their own initiative, since it was set up without the support of health executives or doctors. One NM1 technologist put it this way: “*We settled this between us”*. Ultimately, this workplace organization instigated by paramedical personnel in nuclear medicine units is based on the adoption of tacit rules and shared standards of behavior within the professional group. Job rotation appears to be a collective health preservation strategy (28), enabling stakeholders to carry out their healthcare missions while protecting themselves from radioactivity. By allowing the doses of exposure to radioactivity that are inherent to the care activity to be distributed, the adaptation allows nuclear medicine paramedics to provide care to the patient while limiting their exposure to low doses. In the end, this response can be analyzed as a reformulation of radiation protection rules, in forms adapted to the specificities of patient care.

### Contextualized Prioritization Between Patient Care and Radiation Protection

However, the analysis reveals that adaptation alone does not provide a solution to every contradictory situation faced by paramedical actors. Situations still arise in which contradictions cannot be managed by adapting radiation protection to the treatment. Such situations lead paramedical staff in nuclear medicine to operate a contextualized hierarchy, i.e., an arbitration between the logics of patient care and of self-protection, depending on the situation in hand. From that point on, contradictions are managed by the temporary abandonment of one action logic in favor of the other. Arbitration, then, is situation-dependent; it seems that some situations lead paramedical staff to favor patient care (to the detriment of their own protection), while others lead them to favor their own protection (to the detriment of patient care). The contextualized prioritization strategy appears to be based on a benefit-risk type assessment of the situation by paramedical actors. In the field of radiation protection, benefit-risk analysis refers to the principle of justification according to which any activity entailing exposure to radioactivity must be justified by the benefits it procures, in relation to the risks to which it exposes individuals. From this perspective, risk-taking cannot be justified unless there is a quid pro quo. Paramedical staff in nuclear medicine units weigh up the expected benefits of the examination for the patient and the anticipated risk for the conduct of the examination against the potential occupational risks associated with exposure to low doses. The trade-off between the logics of patient care and self-protection results from this assessment of the situation. Contextualized prioritization thus integrates the two action logics, prioritizing them according to the situation.

Our results also highlight the fact that this contextualized prioritization takes into account various situational parameters such as: patient state of health, type of medical procedure and occupational exposure to radioactivity. We must also point out that the benefit-risk analysis underpinning contextualized prioritization refers to temporal issues that render arbitration more complex. The hierarchy operated by paramedical staff balances a dual temporality that is linked to benefit-risk assessment. Indeed, the benefits and risks for the patient examination are of short-term temporality, whereas the possible occupational risks related to the accumulation of doses of exposure to radioactivity are of long-term temporality.

Ultimately, it seems that the contextualized prioritization implemented by nuclear medicine paramedical personnel gives rise to two types of situations.

- Type 1 situations: actors favor care over protection against the risks associated with low doses where they consider that either the benefit of the examination (or the immediate risk for the examination) to be greater than the long-term occupational risk.
- Type 2 situations: actors favor their protection over care where they consider that the long-term occupational risk associated with low doses to be higher than either the benefit of the examination or the immediate risk for the examination.

For paramedics to prioritize patient care over their own protection, the situation must present an immediate risk to the examination. Thus, where relatives' involvement in the care activity fails to guarantee the proper conduct of the examination, paramedical actors may have to take over from the relatives at the expense of their own protection, as shown in the observation sequence below. This prioritization allows actors to manage the cognitive dissonance they are confronted with by temporarily favoring the logic of patient care—to the detriment of the logic of radiation protection.

**Observation NM1**

A technologist positions an 18 month-old child under the ECAM gamma camera. The child, suffering from neuroblastoma, is on a drip. The mother is also present in the examination room. To prevent the child from moving during the scintigraphy, the technologist straps her to the gamma camera table at leg and chest. Once the child is positioned under the camera, the technologist adjusts the height of the detectors and then asks the mother to hold her child's head still during the examination. The technologist then returns to the control room, starts the examination, but says, a few minutes into the process: “*She moved her head”*. The technologist then returns to the examination room.

- Technologist (to the mother): “*Do you want me to hold her head?”*
- Mother: “*Yes, please.”*

The technologist then places both hands on the child's face to prevent her from moving her head, while the mother sits on a stool next to her child, holding her hand. Paramedical staff may also be led to favor the logic of patient care at the expense of their own protection when they consider that the patient's difficulties in carrying out the tasks requested of them risk jeopardizing the progress of the medical procedure. For example, anticipation of possible patient movements may lead paramedics to stay with the patient throughout the examination, as highlighted in the following observation sequence. Where the patient is unable to perform the tasks required of them, this may lead health professionals to engage in additional work. In the sequence presented below, prioritization is based on taking into account the situational parameters—in particular the patient's state of health.

**Observation NM2**

As part of a myocardial scintigraphy, a nurse has just positioned a patient for the CCAM gamma camera. As the nurse is about to return to the control room to start the examination, the patient, obviously worried, asks “*Will you be nearby?”*, to which the nurse replies: “*Yes, I'm next door”*. The nurse returns to the control room, starts the examination and then addresses one of the technologists in the room: “*This lady might move. She has memory problems, she may forget that I told her not to move”*. The nurse then returns to the examination room and stands next to the patient, at her head. The nurse remains with the patient until the end of the scintigraphic examination.

The existence of an immediate risk for the examination seems to be a necessary (though not stand-alone) condition for understanding the prioritization of patient care over self-protection in certain circumstances. Indeed, prioritization also results from a benefit-risk analysis in which the expected benefit of the examination is weighed against the occupational risk associated with exposure to low doses. As the following verbatim accounts highlight, the actors therefore momentarily favor the logic of patient care over their own protection, where they consider the benefits of the examination for the patient to outweigh the occupational risk: “*I have done brain scans on patients with dementia and almost had to lie down on them to keep them from moving. Because the exam really needed to be done, so right now the irradiation – never mind. Well, you try to put on a lead apron beforehand, but if you're spending 45 minutes with it, holding the patient like that, irradiation, okay, but the patient must have their examination and it must be interpretable. So, you try to do everything, even if it means taking a bit higher dose than usual”* (Technologist NM2).

In addition, situations in which actors favor patient care over protection are also related to exposure to low doses in the long term. Indeed, since the occupational risk comes more from the accumulation of doses than from one-time exposure to radioactivity, these situations allow a hierarchy in favor of patient care precisely because they occur only sporadically in the work activity. As the following verbatim accounts illustrate, these are situations of contradictions related to the clinical and social characteristics of patients, or to certain particularly radiant medical procedures whose frequency of appearance is not daily. “*There have also been a few cases of brain scans, since these patients are a little disturbed anyway, we had to stay with them to talk to them, to keep them company. Since they must not move and it takes a long time, it has happened that we have to stay beside them, but it's still highly unusual”* (Technologist NM2).

However, paramedical personnel in nuclear medicine units do not *systematically* prioritize patient care over their own protection. Indeed, the weighing up of patient benefits against occupational risks can also lead actors to favor their own protection, to the detriment of patient care. This analysis of situations proves a deciding factor in terms of the nature of the hierarchy between the logics of patient care and self-protection, as the following verbatim account shows: “*You have no choice but to let them [the patient] wriggle, and then the images will not be interpretable and they'll never get their examination. If the doctor tells you that the stakes are high for the patient, you'll take the irradiation. However, if the doctor says: “Well, there's nothing else we can do, they move every time”, well, you say to yourself, then I'm not going to… I'm going to let them move, and the exam will be a failure, and they won't get their exam.”* (Technologist NM2).

Paramedical staff therefore prioritize their own protection at the expense of patient care when the occupational risk of the situation is greater than the benefit of the examination for the patient. Thus, insofar as it constitutes a potential source of risk, occupational exposure to radioactivity is not justified if the anticipated benefit of the examination is low or non-existent. The following verbatim account reflects this form of prioritization in the working practices of nuclear medicine paramedical actors: “*I don't know if we would stay to hold an adult, if we can't, we can't. In the end, we are right beside them for their safety, but if they move in all directions, the examination will not be possible. You can't hold a restless patient under the camera”* (Technologist NM2).

Beyond situations in which paramedics consider the risk associated with low doses to be higher than the expected benefit of the examination for the patient, it appears that these actors also favor their own protection over patient care when their analysis of the situation leads them to conclude that the occupational risk is higher than the immediate risk for the examination. Thus, as the following sequence of observations shows, this hierarchy allows nurses and technologists to manage the contradictions between the logics of patient care and of radiation protection by temporarily promoting their own protection, to the detriment of patient care.

Prioritization of the logic of self-protection must also be part of a long temporality that reaches beyond the immediate temporality of the situation. As the following sequence of observations shows, certain situations can lead nuclear medicine paramedics to protect themselves at the expense of patient care, where they anticipate an accumulation of low-dose exposure related to their patient care activity.

**Observation NM2**

In the SYMBIA examination room, a brain scan is underway. The patient keeps calling out to the technologist, who is behind the leaded screens. The technologist goes to the patient, but says that she cannot stay with them during the examination, adding “*You are not alone”*. The technologist then returns behind the leaded screens, explaining that she cannot expose herself too much for one patient, because she has to see several every day. Finally, it seems that the question of the accumulation of exposure doses also allows paramedical personnel to manage the contradictions between the expressive-communication dimension of patient care, and self-protection. Indeed, as the following verbatim account illustrates, the actors justify their own protection to the patient by reference to the repeated nature of the exposure to radioactivity, distinguishing it from the occasional exposure with which patients are confronted in the context of nuclear medicine examinations and treatments: “*Sometimes we have patients who are claustrophobic, dreading the examination, and yet we cannot stay with them. We explain to them that we are continuously exposed to radiation, so we can't hold their hands, be with them for the duration of the imaging. That's why we can't stay too close to the patient, we explain to them that it's because we are exposed all year round. It is still important for us not to increase the dose”* (Technologist NM1).

## Conclusion

The nuclear medicine sector is the subject of little social science research, particularly on the issue of managing the risks of exposure to low doses of radioactivity. This case is all the more interesting because it reflects a situation of uncertainty, in which the logic of precaution is imposed on health professionals. In this particular context, it was interesting to investigate both the relationship to this hypothetical risk and the articulation between risk representations and work practices.

In this respect, this research shows that radiation protection plays an important role in nuclear medicine practice, but that this prevailing precautionary logic is perceived differently. On the one hand, the identified occupational risk management practices reflect a differentiated relationship to the risk of exposure to low doses according to occupational group. On the other, unless we focus on the characteristics of the work activity in nuclear medicine, which is marked by potentially-conflicting patient care and radiation protection requirements, it is difficult to understand why some professional groups react differently to certain situations, deviating from prescribed patient care and radiation protection standards. Risk management practices form part of the work of health professionals, which entails constantly building a compromise between contradictory requirements, and adjustments to both radiation protection rules, and specific situations.

This research makes it possible to identify conflicting logics of action between patient care and occupational radiation protection that challenge the practical intelligence of health professionals and solicit their creativity in managing these arbitration situations. The results highlight the fact that, beyond the prescribed rules, the work activity (which leaves a margin of autonomy) offers professionals (particularly nurses and technologists) the option of resolving these conflict situations that are consubstantial with the work activity in nuclear medicine. The risk management procedures implemented emphasize that the precautionary approach is integrated into the professional practices of the most exposed carers and is based on a temporal assessment of exposure in the short and medium term. The procedures rely on the adoption of tacit rules and shared standards of behavior within nuclear medicine units. Risk management is conducted via a joint distribution of risk among technologists and nurses, and via a vertical distribution of risk between paramedical staff and doctors.

From a methodological point of view, investigating work practices by observation makes it possible to show, beyond normative discourse, that the logic of action has its own dynamics; practice is not entirely regulated in advance by radiation protection measures. Work activity carried out in the context of exposure to low doses of radioactivity results from an adaptation of procedures to the singularity of concrete situations. Yet the activity plays out in the interaction between health professionals, the patient and family members, and this makes it possible to highlight both the collective and situated dimension of risk management.

Given the problem addressed and our choice of methodology (qualitative research), the results are not universal in scope; we remain bounded by contexts and situations. It is thus a matter of what Yin calls theoretical generalization: “analytic generalizations depend on using a study's theoretical framework to establish a logic that might be applicable to other situations” [(11), p. 18]. The aim is to understand what types of representations and mechanisms are at work in a nuclear medicine department, and report on actors' behavior in relation to an unproven risk.

Our results, particularly those highlighting a differentiated relationship to the risk of exposure to low doses between doctors and preparers, concur with those of Zonabend (29) on nuclear workers at La Hague, who shows that while the risk is relativized verbally, workers' practices reveal both a more complex relationship to risk and differentiated forms of collective management. This relationship to differentiated risk can be explained by cultural and identity determinants (30). By highlighting the role of social structures in the construction of risk representations, Douglas' theoretical approach puts the plurality of risk relationships into perspective. This helps explain the relationship between individuals and risks by linking their behavior to the culture of the group to which they belong: a culture characterized by values and beliefs that constitute an implicit frame of reference, mobilized by individuals in their interactions. This work shows the importance of the flexibility available to groups in collectively interpreting and managing risk. It helps explain the relationship between individuals and risks by linking their professional practices to the culture of the group to which they belong. Finally, it shows that although perception of risk is embedded in social structures and contingent contexts, its mobilization also constitutes an identity resource determined by the nature of socio-professional relations, as Zonabend (29) has also demonstrated.

## Data Availability Statement

The datasets generated for this study are available on request to the corresponding author.

## Ethics Statement

This research was carried out within the framework of the IRON Labex (ANR-11-LABX-0018-01). Ethics approval was not required for this study as per applicable institutional and national guidelines and regulations. The informed consent of the participants was implied through survey completion.

## Author Contributions

RL: framing the research, qualitative study, and writing the paper. BG: framing the research and writing the paper. FB: framing the research.

### Conflict of Interest

The authors declare that the research was conducted in the absence of any commercial or financial relationships that could be construed as a potential conflict of interest.

## Figures and Tables

**Table 1:** Interviews in nuclear medicine.

**Occupational group**	**Number of interviews**		**Proportion**	
	**NM1**	**NM2**	**NM1**	**NM2**
Nuclear doctor	3	2	75%	66%
Nuclear doctor intern	1	1	50%	100%
Cardiologist	–	2	–	50%
Radio pharmacist	1	1	100%	100%
Technologist	4	2	80%	50%
Nurse	-	2	–	100%
Healthcare manager	1	1	100%	100%
Auxiliary nurse	-	1	–	100%
Medical physicist	1		100%	
Total	**23**		**70%**	
